# Army Drift

**Published:** 1898-07

**Authors:** 


### ARMY DRIFT.

Complaint after complaint reaches us from those engaged in the veterinary service of the army.

Each battery of light artillery provides for a veterinarian and stable sergeant; 144 horses are allotted to a battery.

The farrier provided for in the cavalry is supposed to attend to the ailments of the horses in that service, no veterinarian being provided for.

At Chickamauga, in June, there were nine batteries of artillery, and of those that had horses issued them quite a number were lost; some six to ten to each company. A large number were unfit for service. Influenza, infectious pneumonia, glanders, and periodic ophthalmia have been very prevalent among the army horses.

At Tampa, Fla., very little effort seems to have been made to provide veterinary medical attendance for the horses. The number of deaths have been very heavy. Any evidence of a proper system of inspection was painfully absent. Sick and well were corralled together. No provision for isolation of those suffering with infectious or contagious diseases. Many bore evidence of having been shipped in a wholly unfit condition. Apparently no system of car-inspection or disinfection was adopted in shipping these animals to Southern military posts.

A staff correspondent of the New York *Times*, speaking of the army veterinary service and the need of a more extended corps of veterinarians as commissioned officers with the needful authority for the better control and regulation of the livestock interests of our army service, makes many strong points that must appeal to the taxpayers of the land, upon whose shoulders must fall the burden of unnecessary waste and loss in this department. He asks why a consulting or managing veterinarian is not maintained at the War Department. Attention is called to the reckless expenditure of money in the purchase of animals totally unfit for the service, and points to the late war and its terrible losses from infectious and contagious diseases. Our losses in times of peace are quoted at 30 per cent.; European armies under proper government veterinary inspection, 10 per cent. The incomplete character of veterinary supplies, the crude medicine-list, the absence of up-to-date methods and facilities for treatment, are all to be noted in our armies. As a civilian, the veterinarian cannot render the government the full service he is capable of, and so brief is his authority that he is powerless to stem the present tide of losses.

The regiment is unfortunate in one thing, and that is the large number of horses on sick-report. Distemper, pink-eye, stiffening in the limbs, are very prevalent among them. It would seem that the government acted very shortsightedly in not providing a veterinary surgeon for each cavalry regiment. The loss in the number of animals already recorded throughout the army, and especially among the cavalry regiments in the South, it is estimated would pay the salaries of a good veterinary surgeon for each regiment for the next two years. There is a movement among the commanding officers of the cavalry regiments to petition Congress to enact a law providing for veterinarians, and it is earnestly hoped that the appeal will not be in vain.—*Black Hill Press.*

The need of properly trained veterinarians to direct the unloading of the horses in connection with General Shafter's invading army was wofully evident, and the loss from injury and drowning were uncalled for in these times when competent and trained men are ready for the army service when proper rank is accorded.

Our Secretary of War has found the aid of Secretary Wilson, of the Department of Agriculture, of the greatest assistance in dealing with questions involving the livestock and rations of the horses and mules. Secretary Wilson fully appreciates the value of the services of veterinarians in his department, clothed with proper authority, and his aid to the Secretary of War must be a forceful reminder to Secretary Alger that there must be commissioned veterinarians in his department, if he is to avoid much just criticism for the suffering and unnecessary losses in his department. Good veterinarians have left the army service because of this lack of recognition. Others will not tender their services until this unjust discrimination against a scientific profession is remedied. Will not Secretary Alger act promptly in this matter?

Will our Government repeat again the imposition of so great a burden upon the livestock owners of our land as it did after the Civil War by the sale of hundreds of glandered horses and mules?

It was estimated that on July 1st the United States had invested $5,000,000 in horses and mules, and this sum of money placed in perishable property that was destined to shrink at a frightful rapidity, not to speak of the total losses endangered and the progress of the service retarded owing to a lack of proper competent veterinary service. How much longer will a long-suffering profession be treated in this way? How much longer shall our tax-burdened people allow this wilful waste?

Japan leads us in the recognition of veterinarians in her army service, while our enemy, the Spanish, have all their army animals under the control of commissioned veterinarians.

While our Government has done little for veterinary education in our country, leading institutions like Harvard, University of Pennsylvania, Cornell, Ames, not to speak of such successful institutions as the American, New York College of Veterinary Surgeons, and many others, have recognized the need of higher veterinary education, fully equipping many educated young men for this profession. In several of the leading veterinary departments of universities, this year, where part of the teaching is conducted in the medical and scientific departments, many of the veterinary students have attained the highest markings, outstripping many of the students in the medical department. It is too late a day to set up inferiority in education as a reason for denying this recognition of a long-suffering profession. Our Government owes it to itself to grant what has been gained by the calling in every other walk of life and State and municipal government.

Dr. W. A. Martin, of Battery A, located at Newport News, Va., joins in the protest of the neglect of the horses in the army; insufficient provision for needful medicines and the ignoring of requisition for the same are resulting in a great shrinkage of value of perishable property of our government.

After many losses of horses at Chickamauga, the quartermaster was authorized in July to engage the services of an *experienced veterinarian* for a period of three months at a salary of seventy-five dollars per month. Surely, without the promise of permanency, a commission, or future provision, our army authorities do not expect to obtain efficient services for such compensation.

A. H. Waddell, "late of the British Army Veterinary Department," sends us an argument in favor of establishing a veterinary department in our army such as they have in the British service. He says : "All the great armies of the Old World maintain a very efficient veterinary corps in connection with the mounted branches of their services, and no other element has been found of greater necessity, usefulness, and importance than the one in question. Its importance and absolute necessity are proved by the enormous amount of money saved by the Government yearly, not only in horses alone, but by the health, condition, and efficiency of the horses of the mounted and transport services, which are due entirely to the efficiency of the veterinary officers of this most estimable and necessary department. I venture to predict that if it is found necessary to send a large body of cavalry, artillery, and transport to Cuba, that the mortality among horses will be enormous ; horses not in condition for such service will be sent, and those that are will die like 'fish out of water,' simply from altered conditions and surroundings, and because there will be no properly disciplined, instructed, qualified, and competent veterinary officers to treat them."

A veterinary surgeon of our army writes as follows : "It is useless to go into our well-known situation again, as I know it has been, too often perhaps, brought to the attention of Congress and army authorities, but without results. We are now situated where, as the term has it, we will have to take it in the neck or quit, and the members of this army do not do the latter until they are counted out for good. A number of us are married, and, in some instances, have families ; in case a Spanish bullet carries one of us off there is no provision made for those we leave behind, although the last raw recruit joining has such protection ; should we, on the contrary, have the good luck to accomplish some brave act worthy of recognition, we should still remain as we are, half citizen and half soldier, and without any definite place or rank in the regiment, as there would be nothing from which to make a start ; we have struggled along in this manner for years, some of us for over thirty, hoping against hope that something would be done to put us on a definite basis. And now that we are about to stake our lives and health, we for the last time hope and ask that Congress will give us a recognized standing and one commensurate with our education, training, and intelligence, and ask the assistance of your influential and recognized paper in our behalf.—*Army and Navy Journal*, July 9, 1898.

